# CRTrack: Low-Light Semi-Supervised Multi-Object Tracking Based on Consistency Regularization

**DOI:** 10.3390/jimaging12090440

**Published:** 2026-09-13

**Authors:** Zijing Zhao, Jianlong Yu, Lin Zhang, Shunli Zhang

**Affiliations:** 1 School of Software Engineering, Beijing Jiaotong University, Beijing 100044, China; 21112202@bjtu.edu.cn (Z.Z.); 24121438@bjtu.edu.cn (J.Y.); 2 School of Information Science and Engineering, Shandong Normal University, Jinan 250014, China; zhanglin20@sdnu.edu.cn

**Keywords:** multi-object tracking, low-light, semi-supervised learning, consistency regularization

## Abstract

Multi-object tracking (MOT) in low-light environments presents significant real-world application value. Despite substantial progress in MOT, low-light MOT remains constrained by the scarcity of specialized datasets, largely because collecting and manually annotating low-light tracking data is difficult and often prohibitively expensive. This paper specifically addresses these challenges through methodological and dataset innovations. We first present low-light multi-object tracking (LLMOT), the first comprehensive low-light MOT dataset containing 11,580 images, including 5316 labeled and 6264 unlabeled images, consisting of nighttime-enhanced MOT17 sequences and multiple unannotated low-light videos. To simultaneously alleviate the constraint of annotation costs and address the damage that low-light-induced image degradation causes to pseudo-label quality, we propose Consistency Regularization Track (CRTrack), a semi-supervised framework tailored for low-light scenarios. Specifically, we introduce a consistent adaptive sampling assignment mechanism that calibrates and filters noisy and shifted pseudo-bounding boxes under low-illumination conditions. We then design an adaptive semi-supervised network update strategy that enables the model to more stably exploit unlabeled low-light videos for iterative optimization. Extensive experiments on the LLMOT dataset validate the effectiveness and robustness of the proposed method. CRTrack achieves 62.472 HOTA, 71.544 MOTA, and 75.864 IDF1 on the LLMOT dataset, demonstrating its effectiveness in low-light MOT. Our approach provides a practical solution for low-light MOT tasks with significant real-world implications.

## 1. Introduction

MOT is a fundamental mid-level task in computer vision [1]. By analyzing video sequences, MOT aims to localize multiple objects and associate them across frames to generate individual tracks [2]. It finds extensive applications in fields such as action recognition [3,4], video analytics [5,6,7], elderly care, and human–computer interaction. In recent years, the practical deployment of MOT has garnered increasing attention, further advancing its development. However, most existing methods are designed to process high-quality input data, often overlooking the low-light conditions commonly encountered in real-world scenarios. Low-light environments are not only ubiquitous in practical applications but also critical in determining the reliability of essential tasks, such as nighttime video surveillance, autonomous driving, and all-weather human–computer interaction. Consequently, advancing research on MOT under low-light conditions is crucial, as it not only addresses the limitations of existing methods but also improves their robustness and adaptability in complex real-world environments.

**Figure 1:**
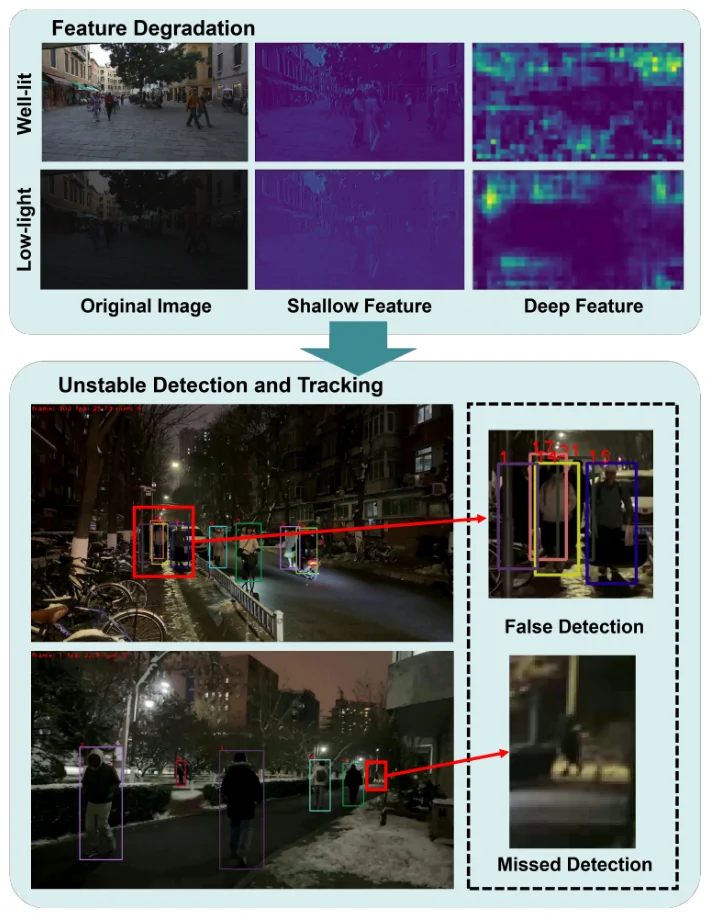
Illustration of the challenges in low-lit multi-object tracking. Compared with well-lit scenes, low-light images produce weaker and less discriminative feature responses, leading to unstable detection and association.

Despite its importance, existing methods face considerable challenges arising from both data limitations and algorithmic constraints. On the one hand, supervised learning approaches heavily depend on large-scale annotated datasets, yet annotated data specific to low-light conditions is severely lacking. The low brightness and high noise inherent in such environments obscure object boundaries, making the generation of high-quality labels both difficult and costly, thereby restricting the availability of suitable training data. On the other hand, algorithms trained on normal-light datasets often fail to generalize effectively to low-light scenarios due to the substantial differences in data characteristics. Specifically, low-light data typically exhibits higher noise levels and reduced contrast, which hinders feature extraction and degrades overall performance. Consequently, these algorithms frequently suffer from missed detections and false positives, as illustrated in Figure 1.

Regarding the physical constraints in data acquisition, videos captured by existing cameras under low-light conditions often exhibit suboptimal quality [8,9]. To avoid motion blur, cameras must shorten exposure times, thereby limiting the amount of light collected. Meanwhile, in order to capture more light signals in low-light conditions, cameras typically increase the gain of the image sensor to enhance image brightness. However, this increase in gain not only amplifies the light signals, but also simultaneously amplifies noise in the image. Such noise obscures image details, resulting in grainy or blurry visuals, which further degrades image quality. Additionally, the low contrast in nighttime environments reduces the dynamic range of the image, making it difficult to preserve details in both bright and dark areas, thus causing further loss of detail. These factors make it difficult for low-light videos to achieve the same level of clarity and detail as daytime recordings.

To address the scarcity of low-light MOT data, we propose a low-light MOT dataset named LLMOT, specifically designed for pedestrian tracking in such environments. The dataset consists of two components: unannotated data and augmented annotated data. The unannotated data, comprising 6264 low-light frames obtained through web crawling and real-world video recordings, aims to capture the diversity and complexity of real low-light scenarios, reflecting the characteristics of actual environments and providing diverse samples to support model generalization. The annotated data is derived from the MOT17 dataset [10] through an augmentation process that simulates nighttime conditions to produce low-light versions of the annotated data. This choice leverages a widely used pedestrian MOT benchmark with high-quality tracks and standardized evaluation protocols, enabling direct comparison with prior detection-based trackers. In contrast, many alternatives either object different settings (e.g., extreme crowd density), lack comparable public annotations, or introduce domain shifts that would confound our goal of isolating the effect of low-light degradation. By integrating these two components, the LLMOT dataset provides both standardized annotated data for controlled evaluation and diverse unlabeled data for semi-supervised learning.

At the algorithmic level, low-light environments impose dual challenges on MOT. Firstly, the collection and annotation of datasets under low-light conditions face significant difficulties. Low brightness and high noise blur object boundaries, making it difficult to distinguish objects from the background, directly increasing the difficulty of obtaining high-quality data and annotations. This limits the construction of large-scale datasets and results in a severe scarcity of high-quality tracking datasets under low-light conditions, particularly MOT datasets targeting pedestrians. Secondly, MOT under low-light conditions presents technical challenges. Existing MOT methods mostly rely on detection-based frameworks, which first detect objects and then associate them across frames [11,12,13,14,15,16,17]. However, these methods depend on high-quality annotated data for supervised learning, and the annotated data under low-light conditions is both scarce and of poor quality, which fails to meet the requirements for model training. Additionally, directly applying pretrained tracking algorithms to low-light scenarios often yields unsatisfactory results due to significant differences in lighting conditions. Some methods attempt to enhance visual quality by incorporating low-light restoration modules [8,18,19], but this significantly increases computational costs and still lacks robustness in complex low-light environments.

In this context, semi-supervised algorithms offer a promising solution for low-light MOT by combining labeled and unlabeled data. These methods alleviate annotation scarcity and improve model generalization in low-light scenarios by exploiting the latent information in unlabeled data. Compared to fully supervised approaches, semi-supervised methods are more data-efficient and adaptable to challenging conditions. While semi-supervised learning has been successfully applied to various fields [20,21], its application to low-light MOT remains underexplored. This highlights the potential value of introducing semi-supervised techniques into low-light tracking tasks as a direction for future research.

Building on the LLMOT dataset, we propose a semi-supervised framework named CRTrack to address the challenges of low-light MOT tasks. CRTrack adopts a “teacher–student” framework based on consistency regularization. The teacher network generates pseudo-labels, and consistency regularization aligns the student network’s predictions with the pseudo-labels, improving the utilization of unlabeled data and enhancing model generalization. To further improve pseudo-label quality, we introduce an adaptive sampling strategy that dynamically selects high-confidence pseudo-labels and optimizes their assignment, reducing the interference of low-quality pseudo-labels. Additionally, we design an adaptive network update mechanism that evaluates network performance during training and prioritizes the better-performing network for pseudo-label generation, further enhancing overall model performance.

In summary, our main contributions are as follows:

- We present the first low-light MOT dataset specifically for pedestrians, which includes a large number of unannotated nighttime pedestrian videos and labeled nighttime data obtained by enhancing the MOT17 [10] dataset.
- We propose CRTrack, a low-light MOT method based on a “teacher–student” network framework, which is capable of leveraging both labeled and unannotated data for semi-supervised training in low-light scenarios. CRTrack improves pseudo-label quality via consistency-based adaptive sampling and assignment, and stabilizes training with an adaptive network update strategy that better exploits unlabeled low-light videos.
- We conduct a comprehensive analysis of the proposed dataset and method. Experimental results demonstrate the effectiveness of our method, highlighting its superior performance and competitiveness in tracking tasks under low-light conditions.

## 2. Related Work

### 2.1. Low-Light Vision and Tracking

Low-light environments pose substantial challenges to visual perception and tracking systems due to insufficient illumination, increased sensor noise, reduced contrast, and image blur. These degradations can obscure object boundaries and weaken visual representations, thereby affecting both object detection and subsequent data association. Existing studies on low-light vision have mainly focused on improving image quality through enhancement and restoration techniques [8,18,19]. Although these methods can improve the visual quality of low-light images, their effectiveness for downstream multi-object tracking remains limited, particularly when the enhanced images still contain noise, blur, or illumination inconsistencies.

In low-light tracking scenarios, the degraded image quality directly affects the reliability of detection and appearance features. Motion-based tracking methods may suffer from inaccurate detections and unstable localization, while appearance-based methods can be sensitive to illumination and visual degradation. Some studies attempt to improve tracking performance by incorporating low-light restoration or enhancement modules [8,18,19]. However, these approaches may introduce additional computational costs and do not fully address the scarcity of annotated low-light tracking data.

Overall, existing low-light vision and tracking studies mainly focus on improving image quality or adapting existing tracking algorithms to degraded visual conditions, while dedicated datasets and data-efficient learning strategies for low-light MOT remain limited. In particular, the lack of annotated low-light pedestrian tracking data makes it difficult to develop and systematically evaluate tracking methods under realistic low-light conditions. These limitations motivate further investigation into dedicated low-light MOT datasets and learning strategies that can effectively exploit both labeled and unlabeled low-light data.

### 2.2. Semi-Supervised Object Detection

Most object detection methods rely on large-scale and well-annotated datasets to achieve cutting-edge performance. However, the high cost of acquiring labeled data drives the development of semi-supervised learning (SSL) methods, which leverage unlabeled data to significantly improve model performance [22,23].

Consistency regularization is among the most widely used techniques in SSL and requires models to produce consistent predictions across augmented versions of the same input [24]. Expanding on this concept, numerous studies optimize model performance through pseudo-label generation and distribution alignment.

For example, FixMatch [22] introduces a high-confidence pseudo-labeling strategy, retaining only pseudo-labels with sufficient confidence to improve efficiency. FlexMatch [25] dynamically adjusts thresholds for different classes to accommodate varying learning difficulties. LaplaceNet [26] leverages graph structures to optimize pseudo-label generation, while DC-SSL [27] aligns predicted distributions with true distributions to improve pseudo-label accuracy. MW-FixMatch [28] addresses class imbalance in SSL by incorporating a weight network to balance contributions from labeled and unlabeled data, which improves performance on imbalanced datasets.

Although these methods enhance model performance on unlabeled data, their effectiveness in specific scenarios, such as low-light environments, remains underexplored. Furthermore, pseudo-label quality is constrained by issues such as confirmation bias, which may lead models to overfit on incorrect pseudo-labels [29]. In low-light environments, image degradation can further increase pseudo-label noise and localization errors, making it more challenging to directly apply conventional pseudo-labeling and consistency regularization strategies. Therefore, low-light MOT requires semi-supervised learning mechanisms that can improve the reliability of pseudo-labels while effectively exploiting unlabeled low-light data.

### 2.3. Multi-Object Tracking

Current MOT algorithms primarily focus on improving tracking algorithms, with less attention paid to data-related issues and diverse application scenarios. Detection-based tracking (DBT) is one of the most widely adopted approaches for pedestrian tracking, detecting objects and performing data association across frames. DBT methods can be broadly categorized into motion-based, appearance-based, and motion-appearance combined approaches.

Motion-based association methods predict object tracks over time to match detections across frames. The classic SORT [11] algorithm uses Kalman filtering and IoU for object matching. Subsequent methods such as BoT-SORT [30] enhance robustness to camera motion, while ByteTrack [15] focuses on improving low-confidence object association. MDATrack [31] further improves motion-based tracking by integrating dynamic motion modeling with adaptive data association, enhancing robustness in challenging conditions. Nevertheless, the performance of these methods tends to degrade in scenarios characterized by object loss or occlusion.

Appearance-based association methods use visual features to match and track objects based on cues such as color, texture, and shape. QDTrack [32] employs contrastive learning to improve object association accuracy without relying on motion priors. FDTA [33] focuses on learning more discriminative object embeddings for association and strengthens identity matching by refining feature representations from detection to association. Li’s work [34] uses multi-scale fusion and Siamese training on discontinuous frames to enhance feature representation and improve association accuracy. DeAOT [35] further improves temporal tracking by decoupling object-agnostic and object-specific representations, enabling more effective propagation of object information across frames. HQTrack [36] further enhances tracking quality through high-quality object representation and refinement strategies, demonstrating strong performance in challenging visual tracking scenarios. However, the sensitivity of appearance-based methods to lighting and pose changes can limit their applicability in degraded visual conditions.

Both motion-based and appearance-based approaches have limitations when applied independently, particularly in handling occlusions and complex motion scenarios. Combined motion-appearance methods integrate motion and appearance cues, yielding better performance in handling occlusions and complex motion patterns [37]. DeepSORT [12] combines appearance features with motion cues, while FairMOT [13,38] unifies detection and re-identification tasks to improve both accuracy and real-time efficiency. FeatureSORT [39] further strengthens online DBT pipelines by optimizing the integration of multiple appearance features, improving robustness under occlusion and reducing identity switches. MMCATrack [3] further explores multi-modal cue fusion and introduces a multi-modal channel attention design to strengthen cross-modal feature aggregation for more reliable association. DetTrack [14] uses spatiotemporal features to mitigate the negative effects of occlusion. GeneralTrack [40] introduces a “point-wise to instance-wise relation” framework, enhancing generalizability across diverse MOT scenarios by improving object association robustness. SVAMOT [41] further explores tracker fusion by exploiting the complementary characteristics of multiple trackers through voting and learning-based scheduling, achieving strong performance on both MOT17 and MOT20 benchmarks. Additionally, unified cue integration methods [42] enhance performance in crowded and occlusion-heavy scenarios. These approaches leverage weak cues (e.g., confidence, pseudo-depth, and height) to compensate for failures of strong cues and refine detection associations, improving overall robustness.

Despite significant algorithmic advancements, DBT methods remain constrained by detector performance, which is inherently affected by the quality and scale of annotated datasets. Collecting and annotating pedestrian tracking datasets is particularly challenging due to large deformations, frequent occlusions, and numerous small objects. For nighttime pedestrian datasets, noise and blur further complicate data collection and reduce data quality. Consequently, most existing MOT methods are developed and evaluated under normal-light conditions, while their robustness can degrade substantially in low-light environments.

To address the limitations of relying on large-scale annotated data, some tracking algorithms have incorporated semi-supervised learning (SSL) techniques to reduce dependence on labeled datasets and enhance performance. These methods combine labeled and unlabeled data, leveraging self-supervised information generated during tracking to optimize models. For instance, some algorithms utilize track information to iteratively train classifiers and extract valuable samples from unlabeled data, achieving accuracy comparable to fully supervised methods even in scenarios with limited labeled data [19]. Furthermore, multi-classifier systems that integrate supervised and semi-supervised models effectively handle challenges in complex scenarios, such as occlusions or visually similar objects, while significantly reducing the risk of object drift [43].

In tracking tasks, SSL techniques are also employed to improve the learning of appearance-based models. For example, some methods use contrastive loss-based embedding mechanisms to extract discriminative appearance features from partially labeled videos, thereby improving tracking robustness [44]. Other approaches analyze the spatiotemporal consistency of object tracks, such as super-track-based video segmentation methods, which group tracks with consistent motion patterns to enable reliable propagation of initial annotations and exhibit stronger adaptability in handling occlusions [45]. However, these methods often rely on track consistency, which can become unreliable in low-light or noisy environments, thereby limiting their reliability and effectiveness in complex scenarios.

Overall, existing MOT methods have achieved substantial progress through motion-based, appearance-based, and combined association strategies. However, most existing approaches are developed for normal-light conditions and rely on reliable detection and appearance cues. In low-light environments, noise, blur, and illumination variations can simultaneously degrade detection quality, feature representation, and data association, while the scarcity of annotated low-light data further limits supervised training. Although semi-supervised tracking provides a potential solution to annotation scarcity, existing approaches may still suffer from unreliable pseudo-labels or track consistency under degraded visual conditions. These limitations highlight the need for a low-light MOT framework that can effectively exploit both labeled and unlabeled data while improving the reliability of learning under low-light conditions.

## 3. Dataset Construction

To address the limitations of existing MOT research in low-light scenarios, we propose a pedestrian tracking dataset specifically designed for low-light environments, named LLMOT. This dataset comprises 11,580 images, including 5316 labeled images and 6264 unlabeled images, offering a comprehensive benchmark to promote MOT research under low-light conditions.

**Figure 2:**
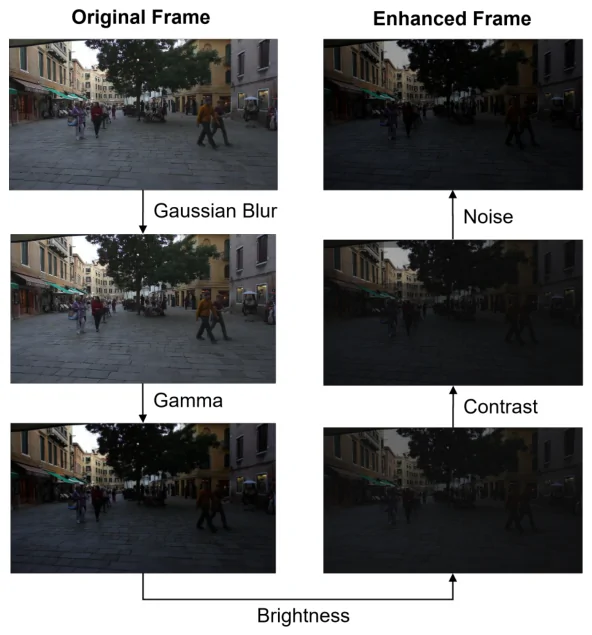
Low-light enhancement results obtained using the manually designed enhancement pipeline, including Gaussian blur and gamma correction.

The labeled data is generated by applying low-light enhancement techniques to seven pedestrian tracking sequences from the MOT17 dataset, simulating diverse nighttime and low-light conditions. To ensure the authenticity and detail preservation of the enhanced images, we evaluated three low-light enhancement methods, including Stable-Diffusion [46], CycleGAN [47], and a manually designed approach. While the Stable-Diffusion model [46,48] excels in image generation tasks, its reliance on the latent diffusion process often sacrifices fine-grained details to emphasize global features [49]. Additionally, the high computational cost of Stable-Diffusion makes it impractical for large-scale dataset construction. Similarly, CycleGAN [47,50] enables effective style transfer, but its adversarial training mechanism lacks temporal consistency constraints, leading to noticeable artifacts in video sequences. Furthermore, its sensitivity to training parameters can degrade performance and visual quality under complex low-light conditions. The related experimental results are demonstrated in Section 5. In contrast, prior work [51] has shown that a manually designed series of enhancement strategies can effectively simulate complex degradations while preserving fine details. Inspired by this finding, we further design a manual enhancement pipeline to generate the labeled dataset, as illustrated in Figure 2. We further validate the realism of the enhanced data in Section 5 through quantitative comparisons with real night-scene datasets.

Low-light images typically exhibit reduced brightness, weakened contrast, and lower signal-to-noise ratio (SNR). Additionally, physical limitations of imaging devices in low-light conditions often result in blurring and noise. The enhancement operations are arranged to mimic the image formation and degradation process commonly observed in real low-light environments. Gaussian blur is applied first to simulate optical degradation caused by limited illumination and camera motion. Gamma correction is then used to reproduce nonlinear illumination changes introduced by camera imaging pipelines. Brightness and contrast adjustments further model variations in scene illumination and visibility. Finally, Gaussian noise is added to emulate sensor noise, which becomes more prominent under low-light conditions. This progressive design enables the generated images to better reflect realistic low-light characteristics while preserving temporal consistency and object details. To accurately simulate these characteristics, we designed an enhancement strategy that includes Gaussian blur, gamma correction, brightness adjustment, contrast modification, and Gaussian noise addition, with randomization applied at each step to create images with greater complexity and diversity. The overall enhancement process can be expressed as

(1) $$I^{\prime }=\mathrm{Noise}\left({\mathrm{Contrast}}_{\alpha }\left({\mathrm{Brightness}}_{\Delta B}\left({\mathrm{Gamma}}_{\gamma }\left(G_{\sigma }\left(I\right)\right)\right)\right)\right),$$

where *I* and $I^{\prime }$ denote the original input image and the enhanced image, respectively. The first operation applies Gaussian blur, denoted by $G_{\sigma }\left(I\right)$, where σ is the standard deviation of the Gaussian kernel. The gamma correction operation, denoted as ${\mathrm{Gamma}}_{\gamma }\left(x\right)=x^{\gamma }$, applies a nonlinear transformation to the image, where γ is a random exponent sampled from $\gamma ∼U(\gamma_{\min},\gamma_{\max})$. After gamma correction, brightness adjustment is performed by ${\mathrm{Brightness}}_{\Delta B}\left(x\right)=x+\Delta B$, where ΔB∼U(−β,β). Subsequently, contrast adjustment is performed by ${\mathrm{Contrast}}_{\alpha }\left(x\right)=\alpha x$, where α∼U(1−λ,1+λ). Gaussian noise is then added to the image according to Noise(x)=x+ϵ, where $\epsilon ∼N(0,\sigma_{n}^{2})$ represents Gaussian noise with mean zero and variance $\sigma_{n}^{2}$. The randomization of parameters ΔB, α, γ, σ, and $\sigma_{n}$ ensures that the augmented images exhibit diverse and complex low-light characteristics. This strategy mitigates the risk of artifacts and over-enhancement while preserving consistency with the natural properties of low-light images, such as brightness, contrast, and the SNR.

The unlabeled dataset consists of 10 video clips captured from real low-light scenarios, containing a total of 6264 frames. These data cover various low-light conditions, such as nighttime scenes, overcast weather, and urban environments with significant occlusions caused by railings or dense crowds. The inclusion of real-world data provides semi-supervised learning methods with rich latent information, enhancing the robustness and generalization capabilities of models under low-light conditions. The labeled data are divided into a training set and a test set at the frame level based on temporal order within each video sequence. Specifically, for each labeled sequence, the first 60% of frames are reserved as the training portion, while the remaining 40% are held out as the independent test set. Within the training portion, 40% of the frames are further held out as a validation set according to the same temporal order, while the remaining 60% are used for model training. The validation set is used exclusively for adaptive network updating and checkpoint selection, whereas the test set is never accessed during training or adaptive network updating and is used only for final performance evaluation. All unlabeled data assigned to the training portion are used for semi-supervised learning.

## 4. Methodology

In this section, we introduce the overall framework of our low-light semi-supervised MOT method, CRTrack, which is built on consistency regularization. The proposed tracking framework consists of two main components: detection for identifying objects in each frame and association for linking these objects across frames. First, we construct a semi-supervised object detection framework based on a “teacher–student” network structure (explained in Section 4.1), which leverages both labeled and unlabeled data to drive the core of the detection process. The semi-supervised detector comprises two key modules: a consistent adaptive sampling assignment mechanism described in Section 4.2 and an adaptive update method described in Section 4.3. These modules are specifically designed to enhance the detector’s performance in low-light conditions. Once trained, the detector is used to accurately identify objects in video frames, serving as the foundation for subsequent tracking. Subsequently, the object association method is improved to more efficiently link detected objects across frames, ensuring temporal consistency and robust tracking (explained in Section 4.4).

### 4.1. Semi-Supervised Framework

In detection-based MOT frameworks, robust detection performance is critical to providing accurate input data for subsequent data association. However, low-light scenarios pose significant challenges due to the scarcity of labeled data and complex illumination conditions, which substantially degrade detection performance. To address the issue of limited labeled data and improve training efficiency by leveraging pretrained models, we propose a semi-supervised learning method based on consistency regularization.

In this framework, the detection process consists of two main components: feature extraction and prediction. Feature extraction aims to learn robust representations from both labeled and unlabeled data, while prediction focuses on accurately classifying objects and regressing their bounding boxes. Consistency regularization is introduced to enhance the model’s ability to learn from unlabeled data by constraining predictions to remain consistent under different perturbations applied to the same input. Accordingly, we propose an illumination-aware consistency regularization strategy within our framework to tackle the challenges posed by varying illumination. By leveraging consistency regularization, the model can learn illumination-invariant feature representations, thereby improving its detection performance and adaptability.

**Figure 3:**
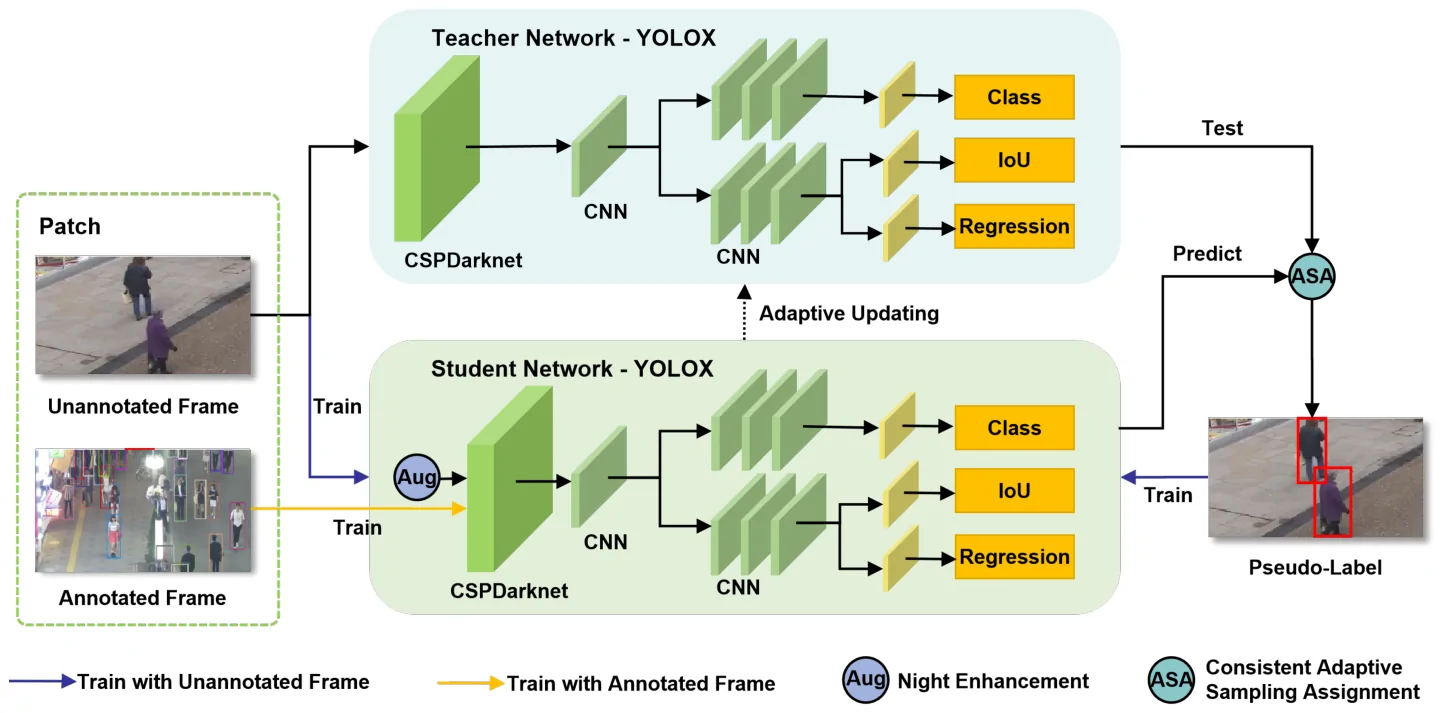
“Teacher–student” network pipeline. The teacher network generates pseudo-labels to guide the student network, while the student network is used to update the teacher network during training.

To tackle the challenges associated with semi-supervised object detection under low-light conditions, we propose a low-light-oriented “teacher–student” network framework. The proposed framework mitigates low-light-induced pseudo-label noise and localization shifts, enabling more stable exploitation of unlabeled data through iterative refinement. We build both the teacher and student networks on YOLOX [52] as the base detector, as illustrated in Figure 3. The teacher network performs object detection and generates pseudo-labels to guide the training of the student network. Through consistency regularization, the student network is encouraged to produce consistent predictions under different perturbation conditions for the same pseudo-label. The weights of the student network are then used to adaptively update and fine-tune the teacher network.

These networks are initialized with identical pretrained parameters. During training, each batch is constructed by combining annotated and unannotated data, which are processed to train the student network.

In a batch, the annotated data is trained using standard supervised learning with the ground truth (GT), while the unannotated data is first processed through the teacher network for object detection, where consistent adaptive sampling assignment (ASA) is applied to dynamically select high-confidence predictions as pseudo-ground truth. These pseudo-labels are then used to train the student network, while the input of the student network is processed using a manually designed nighttime augmentation method. During training, consistency regularization is introduced to improve the model’s robustness under low-light conditions by enforcing the output of the student network, after receiving augmented input, to remain consistent with the output of the teacher network. The teacher network $f_{t}\left(\cdot ;\Theta_{t}\right)$ and the student network $f_{s}\left(\cdot ;\Theta_{s}\right)$ are initialized with the same pretrained weights from a YOLOX detector trained on large-scale annotated data captured under normal lighting conditions. Subsequently, the unannotated data under low-light conditions is used for fine-tuning, further optimizing the network performance by minimizing the training loss on the unannotated data.

To effectively exploit teacher-generated pseudo-labels on unlabeled data and to improve training stability and localization robustness under low-light conditions, we formulate the unlabeled loss $L_{U}$ as a weighted combination of classification, box regression, and IoU terms. Specifically, the classification term constrains category prediction, the regression term provides coordinate-level supervision, and the IoU term further imposes an overlap-based geometric constraint, enabling more robust joint optimization when pseudo-labels are noisy or uncertain. Formally, the loss $L_{U}$ on the unannotated data $C_{U}$ is defined as

(2) $$L_{U}=\frac{1}{M}\sum_{i=1}^{M}[\lambda_{cls}L_{cls}\left(f_{s}\left(E\left(x_{i}\right)\right),{\hat{y}}_{i}\right)+\lambda_{reg}L_{reg}\left(f_{s}\left(E\left(x_{i}\right)\right),{\hat{y}}_{i}\right)+\lambda_{IoU}L_{IoU}\left(f_{s}\left(E\left(x_{i}\right)\right),{\hat{y}}_{i}\right)],$$

where *M* is the number of unlabeled samples in a mini-batch, ${\hat{y}}_{i}=f_{t}\left(x_{i};\Theta_{t}\right)$ denotes the pseudo-label generated by the teacher network, and E(·) represents the proposed nighttime augmentation applied to the student input. The loss weights $\lambda_{cls}$, $\lambda_{reg}$, and $\lambda_{IoU}$ balance the classification loss $L_{cls}$, the box regression loss $L_{reg}$, and the IoU loss $L_{IoU}$, respectively.

During training, the labeled data serves as an accurate baseline, allowing the model to better fit the data distribution. Given a labeled set $C_{L}={\left\{x_{j}\right\}}_{j=1}^{N}$ with *N* frames, the supervised loss is defined as

(3) $$L_{L}=\frac{1}{N}\sum_{j=1}^{N}[\lambda_{cls}L_{cls}\left(f_{s}\left(x_{j}\right),y_{j}\right)+\lambda_{reg}L_{reg}(f_{s}\left(\left(x_{j}\right),y_{j}\right)+\lambda_{IoU}L_{IoU}\left(f_{s}\left(x_{j}\right),y_{j}\right)],$$

where $y_{j}$ represents the ground truth of the *j*th image.

The total loss is calculated as

(4) $$L_{T}=\lambda_{U}L_{U}+L_{L},$$

where $\lambda_{U}$ represents the weight parameter for the unannotated loss, determined by the proportions of annotated and unannotated data in the batch.

### 4.2. Consistent Adaptive Sampling Assignment

In existing methods, the teacher network generates pseudo-labels for student training by retaining predictions whose confidence scores exceed a fixed threshold. However, under low-light conditions, pseudo-labels can be noisy or misaligned, making such static selection unreliable. Consistent-Teacher [53] alleviates this issue with an adaptive assignment mechanism that reduces the influence of noisy pseudo-boxes. However, its formulation is built on anchor-box matching in anchor-based detectors and is therefore not directly compatible with YOLOX’s anchor-free design. To this end, ASA is proposed for YOLOX, enabling adaptive pseudo-label assignment in an anchor-free manner and providing more reliable supervision for semi-supervised training.

In addition, unlike Consistent-Teacher, which is primarily designed for multi-class object detection tasks, our work focuses on single-class pedestrian detection tasks. Multi-class object detection tasks often require separate assignment strategies for each class, while single-class pedestrian detection aims to distinguish pedestrians from the background, simplifying the complexity of pseudo-label assignment but requiring higher precision in the assignment process. Considering the characteristics of pedestrian detection, we design a method that better suits the requirements of single-class pedestrian detection.

Given an object instance *u* in frame *x*, we denote by $x^{u}$ the spatial region corresponding to instance *u*. As the same pseudo-label assignment principle can be applied analogously to classification, objectness prediction, and localization regression, we present the formulation for the bounding-box component for brevity. The instance-level pseudo-box is formulated as

(5) $$\hat{g}=\underset{g}{\arg\min}\;L\left(f_{t}\left(x^{u}\right),g\right),$$

where *g* denotes a candidate pseudo-box and is treated as the optimization variable. The function L(·,·) defines a consistency-based selection cost that measures the discrepancy between the teacher signal $f_{t}\left(x^{u}\right)$ and the candidate *g*. Minimizing L yields the final pseudo-box $\hat{g}$, which therefore best agrees with the teacher prediction among the candidates. In principle, pseudo-boxes are produced by the teacher. Training a dense detector still requires assigning each pseudo-box to a subset of student predictions. A static pseudo-label generation or assignment strategy may amplify pseudo-box noise and introduce noisy supervision.

Accordingly, pseudo-box assignment is performed by estimating the pairwise consistency between dense student predictions and teacher-generated pseudo-boxes. Let the student network produce *N* dense predictions $P^{u}={\left\{p_{n}\right\}}_{n=1}^{N}$ for $x^{u}$, where $p_{n}=f_{s}{\left(E\left(x^{u}\right)\right)}_{n}$ denotes the *n*th prediction obtained from the enhanced input. Here, E denotes the low-light enhancement function introduced in Section 3, and is also used as an additional perturbation in LLMOT experiments. Let the teacher network generate *L* pseudo-boxes $Y^{u}={\left\{y_{k}\right\}}_{k=1}^{L}$.

Rather than treating all high-confidence predictions as positive samples, we select student predictions that show stronger consistency with teacher supervision. Although pseudo-box assignment over dense predictions can be viewed as a discrete prediction-to-pseudo-box matching problem, explicitly solving the global combinatorial assignment is inefficient in practice. Therefore, we compute a pairwise matching cost between each student prediction and each teacher pseudo-box, and derive an efficient approximate assignment by selecting low-cost matches as positive samples.

YOLOX adopts an anchor-free formulation, where each dense prediction naturally corresponds to a candidate object center and its box size. However, if pseudo-box assignment is mainly dominated by classification, regression, and IoU costs, the center consistency between dense predictions and teacher pseudo-boxes is not explicitly enforced. This may lead to unreliable assignments when pseudo-boxes are noisy or slightly mislocalized, especially under low-light conditions. To address this issue, we propose a center-aware matching scheme that augments the matching cost with a lightweight center-distance prior. For a student prediction $p_{n}$ and a teacher pseudo-box $y_{k}$, the matching cost $C_{nk}$ is defined as

(6) $$\begin{array}{cc} C_{nk}= & \lambda_{cls}L_{cls}\left(p_{n},y_{k}\right)+\lambda_{reg}L_{reg}\left(p_{n},y_{k}\right)+\lambda_{IoU}L_{IoU}\left(p_{n},y_{k}\right)+\lambda_{dis}C_{dis}\left(p_{n},y_{k}\right), \end{array}$$

where $L_{cls}$, $L_{reg}$, and $L_{IoU}$ denote the classification, regression, and IoU losses, respectively. The term $C_{dis}$ measures the Euclidean distance between the prediction center and the center of pseudo-box $y_{k}$, serving as a center prior during training. The weighting parameters $\lambda_{cls}$, $\lambda_{reg}$, $\lambda_{IoU}$, and $\lambda_{dis}$ are used to balance different cost terms, where $\lambda_{dis}$ is assigned a small value to avoid overwhelming the original detection costs.

Based on the matching cost $C_{nk}$, predictions with the top-*K* lowest costs for each pseudo-box are assigned as positive samples. If one prediction is selected by multiple pseudo-boxes, it is assigned to the pseudo-box with the minimum matching cost. Predictions that are not selected by any pseudo-box are assigned to the background category, namely $a_{n}=L+1$. Compared with fixed-threshold pseudo-label selection, the top-*K* low-cost matching strategy avoids sensitivity to an absolute confidence or loss threshold under varying noise levels and provides a stable number of positive samples for each pseudo-box. By combining loss-guided filtering with center-aware matching, the proposed ASA method keeps fewer but more reliable pseudo-labels and suppresses noisy supervision.

### 4.3. Adaptive Network Updating

In EMA-based “teacher–student” frameworks for semi-supervised detection, the teacher network provides temporally smoothed objects for pseudo-label generation, which is beneficial for stabilizing training. However, when the student network adapts rapidly, an EMA-only teacher update can introduce a temporal lag, such that the teacher becomes under-adaptive to the student. This lag may yield pseudo-labels that are inconsistent with the student’s current predictions and thus less reliable as supervision signals, which can hinder optimization and amplify training instability. Such “teacher–student” inconsistency has been shown to degrade pseudo-label quality and localization consistency in semi-supervised detection [54]. The problem is further aggravated in low-light scenarios, where reduced contrast and increased noise make the predictions more sensitive and the “teacher–student” mismatch more pronounced.

To prevent the teacher from lagging behind the student while avoiding noisy updates, we propose a validation-guided gated update strategy. Specifically, at the end of each training epoch, we evaluate both the teacher and the student on a validation set using mAP. The validation set is obtained by splitting the labeled training data and is used exclusively for adaptive network updating and checkpoint selection. It is not used for final performance evaluation, which is conducted only on the held-out test set. In addition, we maintain a historically best-performing checkpoint, denoted as bestModel, which stores the parameters achieving the highest validation mAP observed so far and is used for checkpoint selection.

The teacher is updated using a piecewise rule. If the student outperforms the current teacher on the validation set, we perform a hard update by copying the student parameters to the teacher to promptly reduce the “teacher–student” lag. Otherwise, we update the teacher using EMA to smooth training fluctuations and keep the teacher stable. The update rule is defined as

(7) $$\Theta_{t}=\left\{\begin{array}{cc} \Theta_{s}, & \mathrm{if}\;\mathrm{eval}\left(\Theta_{s}\right)>\mathrm{eval}\left(\Theta_{t}\right),\\ \alpha \cdot \Theta_{t}+\left(1-\alpha \right)\cdot \Theta_{s}, & \mathrm{otherwise}, \end{array}\right.$$

where eval(Θ) denotes the validation set performance (mAP) of parameters Θ, and α controls the EMA smoothing strength. This adaptive strategy mitigates the “teacher–student” temporal misalignment while retaining the stability of EMA updates.

The pseudocode of the proposed update method is provided in Algorithm 1.
**Algorithm 1** Adaptive update method  1: bestModel←teacher  2: $bestEval\leftarrow eval_{\mathrm{val}}\left(bestModel\right)$  3: **while** training **do**  4:       Train student for one epoch  5:       $teacherEval\leftarrow eval_{\mathrm{val}}\left(teacher\right)$  6:       $studentEval\leftarrow eval_{\mathrm{val}}\left(student\right)$  7:       **if** studentEval>teacherEval **then**  8:             teacher←student▹ fast alignment  9:       **else**10:             teacher←EMA(teacher,student)▹ EMA smoothing11:       **end if**12:       $teacherEval\leftarrow eval_{\mathrm{val}}\left(teacher\right)$13:       **if** teacherEval>bestEval **then**14:             bestModel←teacher15:            bestEval←teacherEval16:       **end if**17: **end while**18: Evaluate bestModel on the independent test set

### 4.4. Split Cosine-Distance Appearance Association

In low-light MOT, weakened object textures, blurred boundaries, and increased environmental noise often lead to unstable detections and ambiguous data association. In such scenarios, relying only on motion cues is insufficient, particularly when objects are close to each other, temporarily occluded, or frequently missed by the detector. Although appearance features can provide complementary identity information, low-light degradation may make appearance embeddings unreliable and consequently increase the risk of identity switches.

To address these challenges, we propose a low-light appearance-motion association method in CRTrack. The proposed method is designed to improve the robustness of appearance-affinity computation under low-light degradation. The motion branch follows the observation-centric tracking paradigm of OC-SORT to maintain short-term trajectory continuity under missed detections and occlusions, while the appearance branch introduces a split cosine-distance association module to exploit local appearance information for identity discrimination. The appearance embeddings are extracted using FastReID and are used to measure the visual affinity between current detections and existing tracks.

Specifically, for each detection and track, the corresponding bounding-box region is divided along the vertical direction into three horizontal local patches, representing the upper, middle, and lower regions of the object. A normalized appearance embedding is independently extracted from each patch using FastReID. Thus, each detection and track is represented by three local feature vectors. For each detection–track candidate pair, the local features of the detection and track are compared in a pairwise manner, resulting in nine cosine similarities between the three detection-side and three track-side local features. For each track-side local region, only the highest similarity with the three detection-side local regions is retained as its best local correspondence, resulting in three local affinities. A hard local-consistency constraint is then applied to these three local affinities. If any of them is lower than the threshold $\tau_{a}=0.55$, the candidate detection–track pair is regarded as having insufficient local appearance agreement, and its appearance affinity is directly set to zero. Only when all three track-side local regions have a sufficiently similar counterpart in the detection does the candidate pair pass the consistency check. For a candidate pair that passes this check, the maximum of the three local affinities is used as the final appearance affinity.

**Figure 4:**
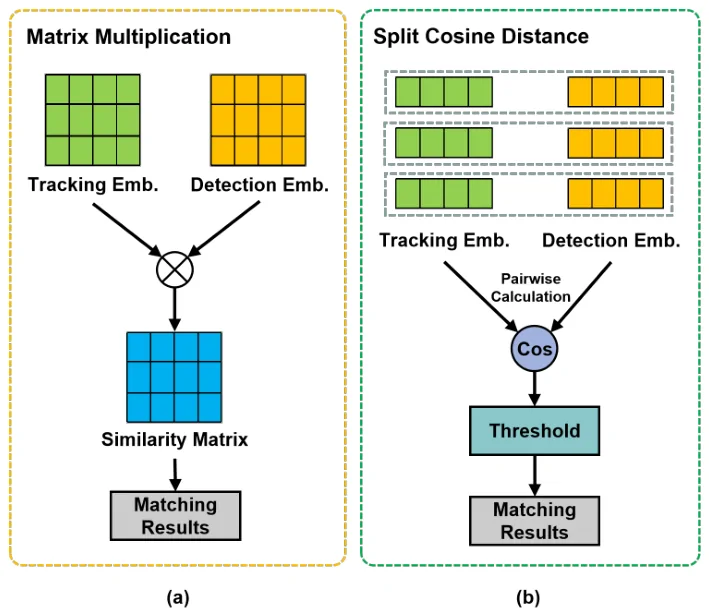
Comparison of appearance association strategies. (**a**) Matrix multiplication is adopted by Deep OC-SORT, and (**b**) the proposed split cosine-distance association with local feature matching and consistency filtering in CRTrack.

Figure 4 illustrates the difference between conventional global appearance association and the proposed split cosine-distance association. Deep OC-SORT computes appearance affinity using a single global appearance embedding for each detection and track. In contrast, CRTrack first divides the target region into three local patches, performs local cross-feature matching, and applies a hard consistency constraint before obtaining the final appearance affinity. This design enables the tracker to exploit local appearance correspondence and suppress unreliable local matches under low-light conditions.

We measure the similarity between local appearance features using cosine similarity, which is defined as

(8) $$\mathrm{Similarity}\left(x,y\right)=\frac{x\cdot y}{\left|x||y\right|},$$

where x and y denote a pair of local feature embedding vectors extracted from the detected object and the track, respectively. x·y denotes the dot product of the two vectors, while |x| and |y| are their Euclidean norms. This formulation measures the directional similarity between the two feature vectors. Since cosine similarity is insensitive to variations in feature magnitude, it reduces the influence of feature-scale variations, which is particularly beneficial for appearance features affected by degradation in low-light environments.

The candidate filtering is performed using the IoU threshold $\tau_{\mathrm{IoU}}=0.3$. The IoU threshold is used to constrain the spatial validity of the final associations, while the appearance affinity and motion cues are jointly incorporated into the association score. This mechanism effectively eliminates spatially implausible associations, thereby improving the robustness of the matching process.

The primary advantages of the proposed split cosine-distance association lie in its ability to exploit local appearance correspondence and enforce appearance consistency across different object regions. By representing each object with three local appearance embeddings rather than a single global feature, the proposed strategy can better tolerate partial appearance degradation caused by low-light conditions, occlusion, or background interference. The local feature matching and consistency constraint further suppress candidate pairs with insufficient local appearance agreement, thereby reducing unreliable associations. By jointly considering spatial overlap, velocity-direction consistency, and appearance affinity, the proposed association strategy effectively exploits complementary cues to improve matching reliability and reduce identity switches in challenging low-light environments.

## 5. Experiments

### 5.1. Experiment Setup

**Figure 5:**
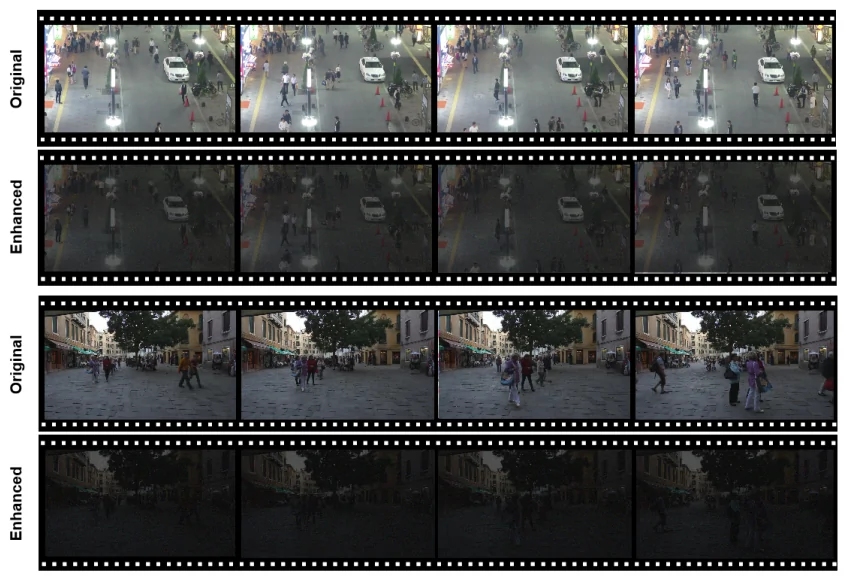
Two example videos from the enhanced MOT17 sequences in the LLMOT dataset. The enhancement introduces darker illumination and increased blur while preserving the original pedestrian locations.

**Figure 6:**
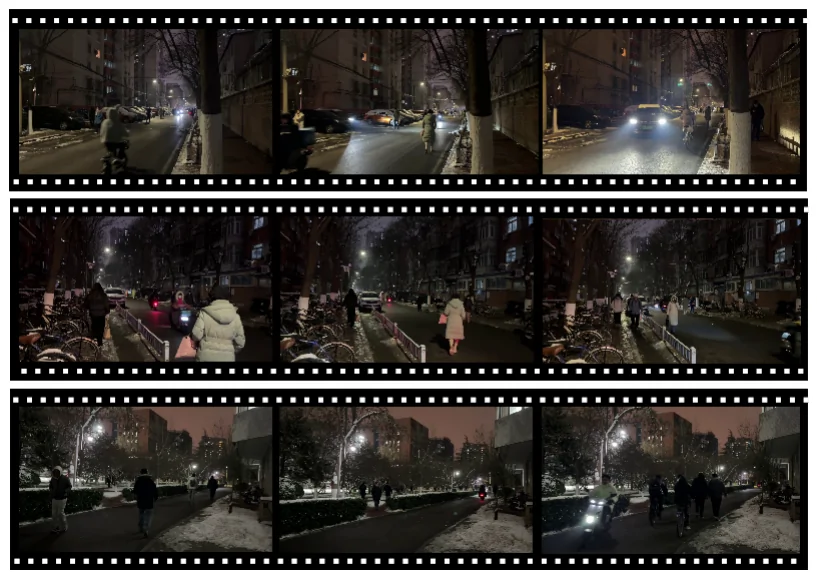
Three example videos of unannotated data from our LLMOT dataset. Each row consists of three frames selected from a video.

**Dataset.** Since this work focuses on low-light multi-object tracking, the LLMOT dataset is adopted as the primary benchmark for our main experiments. LLMOT is specifically designed for low-light RGB-based object tracking tasks. It consists of MOT17 data enhanced to simulate low-light conditions, together with additional unannotated low-light data, as illustrated in Figure 5 and Figure 6. By providing low-light tracking scenarios with degraded illumination, blurred object boundaries, and reduced visual discriminability, LLMOT serves as the core dataset for evaluating the effectiveness of the proposed method under challenging low-light conditions. To further assess the authenticity of the enhanced low-light data in LLMOT, we compare it with two real-world low-light datasets, UniRTL [55] and LLOT [56]. These datasets are collected from real low-light scenes and are used as reference benchmarks for evaluating whether the enhanced data in LLMOT exhibits realistic low-light characteristics. It should be noted that UniRTL and LLOT are not used as the primary tracking benchmarks in our experiments, since they are not explicitly designed for MOT evaluation. Instead, they are employed to support the analysis of the realism and domain consistency of LLMOT. In addition to the low-light datasets used for evaluation, MOT17 and CrowdHuman [57] are utilized only during the pretraining stage to learn generalized pedestrian detection and tracking representations.

**Evaluation Metrics.** To ensure reliable evaluation, the authenticity of the enhanced dataset is first verified by comparing its brightness (BM), noise level (Noise_std), color mean (CM), and SNR with real-world datasets such as UniRTL and LLOT. Brightness (BM) reflects the average intensity of light in an image, capturing the overall illumination levels typical of low-light scenes. Noise level (Noise_std) quantifies the standard deviation of noise in the image, representing the degree of random variations caused by low exposure or high gain settings. The CM measures the average value of color channels (e.g., RGB) in the image, which helps assess whether the color distribution is consistent with real-world low-light environments. The SNR evaluates the relative strength of the meaningful image content to the noise, indicating the overall quality of the image. These comparisons ensure that the enhanced data falls within a reasonable range of real-world nighttime scenes, thereby providing a solid foundation for subsequent evaluations.

Subsequently, object detection and tracking performance are evaluated using several metrics. Detection accuracy (DetA) [58] measures overall detection precision. Multiple object tracking accuracy (MOTA) [59] accounts for false positives, missed detections, and identity errors. Higher-order tracking accuracy (HOTA) provides a detailed analysis of detection quality and track association. The identity F1 score (IDF1) [60] evaluates both detection accuracy and track consistency. Association accuracy (AssA) [58] focuses on the association accuracy between objects and tracks. Combined, these metrics comprehensively assess the performance of the tracking method. Additionally, object detectors are evaluated using average precision (AP) and average recall (AR). Unless otherwise specified, all percentage-based metrics, including DetA, AssA, MOTA, HOTA, IDF1, AP, and AR, are reported in percent (0–100). For brevity, the % symbol is omitted in tables.

**Implementation Details.** We implement CRTrack as a detection-based MOT framework with a low-light semi-supervised detector and our split cosine-distance appearance association fused with motion cues. Both the teacher and student detectors are built on YOLOX-x [52], while the motion association branch follows the Deep OC-SORT module [16]. Initially, YOLOX is trained using supervised learning on the CrowdHuman dataset and the annotated training portion of the LLMOT dataset for 80 epochs. The model is then fine-tuned using semi-supervised learning with the CrowdHuman dataset and the training set of LLMOT for an additional 30 epochs. A validation subset split from the training data is used for adaptive network updating and checkpoint selection, while the independent test set is reserved exclusively for final evaluation. The input resolution is set to 1440×800 pixels. The detector is optimized using SGD with a momentum of 0.9 and a weight decay of $5\times 10^{-4}$. The learning rate follows the YOLOX warm-cosine schedule with a 5-epoch warm-up and a minimum learning-rate ratio of 0.05. Following the YOLOX learning-rate scaling rule, the base learning rate is set to $2.5\times 10^{-4}$, and the per-image learning-rate coefficient is set to 0.001/64. The EMA coefficient is set to 0.9998. The loss weights are set to 5.0 for the IoU loss, 1.0 for the objectness loss, 1.0 for the classification loss, and 1.0 for the L1 loss when enabled. The low-light augmentation parameters are set to β=0.8, λ=0.8, $\gamma_{\min}=1$, and $\gamma_{\max}=5$, while the Gaussian blur parameter and Gaussian noise standard deviation are sampled from σ∈[0.1,2.0] and $\sigma_{n}\in \left[0,50\right]$, respectively. For pseudo-label generation, teacher predictions are retained when the objectness confidence exceeds 0.6 and the class confidence exceeds 0.5, followed by non-maximum suppression with an IoU threshold of 0.65. For appearance-based data association, we use the pretrained FastReID MOT17 SBS-S50 model provided by the Deep OC-SORT implementation to extract appearance features. The model is used without additional training or fine-tuning, and no additional training data are introduced. The appearance-fusion weight is set to 0.75, the adaptive-weighting parameter is set to 0.5, the appearance-affinity threshold is set to 0.55, and the IoU threshold for candidate filtering is set to 0.3. The ASA top-*K* is set to 10. Other parameters inherited from the baseline tracking implementation, including the original Deep OC-SORT configuration, are kept unchanged and are therefore not redundantly repeated in this manuscript. The semi-supervised training process takes approximately 18 h on four NVIDIA RTX 3090 GPUs (NVIDIA Corporation, Santa Clara, CA, USA), with four images processed per GPU, including three labeled images and one unlabeled image, resulting in a global batch size of 16.

### 5.2. Main Results

**Dataset construct.** To evaluate the effectiveness of different low-light enhancement methods for nighttime scenarios, Stable-Diffusion, CycleGAN, and our proposed manually designed method are compared.

**Figure 7:**
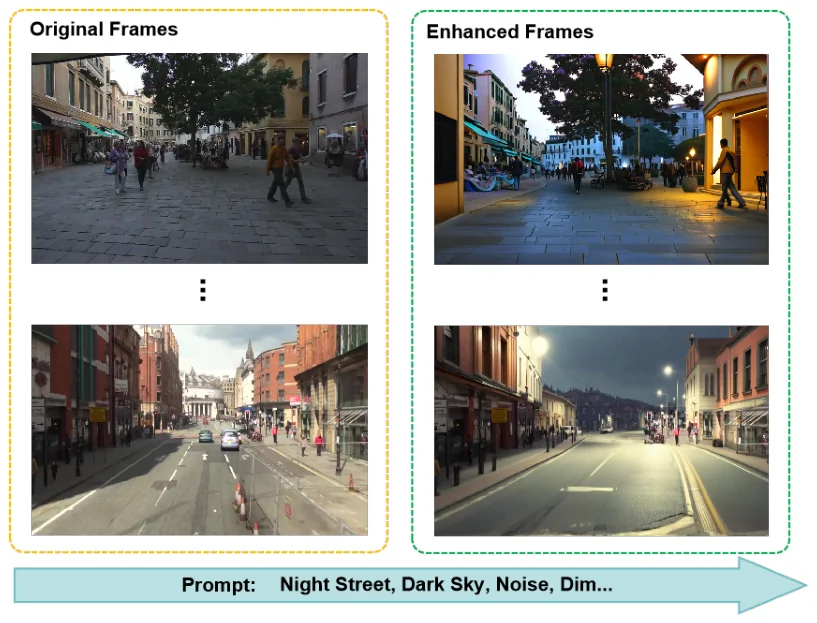
Low-light enhancement results generated using the Stable-Diffusion model. The original image is shown on the left, while the enhanced result is shown on the right using prompts such as “night street,” “dark sky,” “noise,” and “dim.”

The Stable-Diffusion model faces challenges in nighttime enhancement for the MOT17 dataset due to its limitations in preserving fine details and producing physically realistic lighting effects. While capable of global style adjustments, it often blurs or omits key objects such as pedestrians, thereby compromising data integrity. Furthermore, the generated images frequently exhibit inconsistencies in lighting and shadow realism, making them unsuitable for downstream tasks. The computationally intensive diffusion process also limits the scalability of this method for large-scale augmentation. Examples of these issues are illustrated in Figure 7.

**Figure 8:**
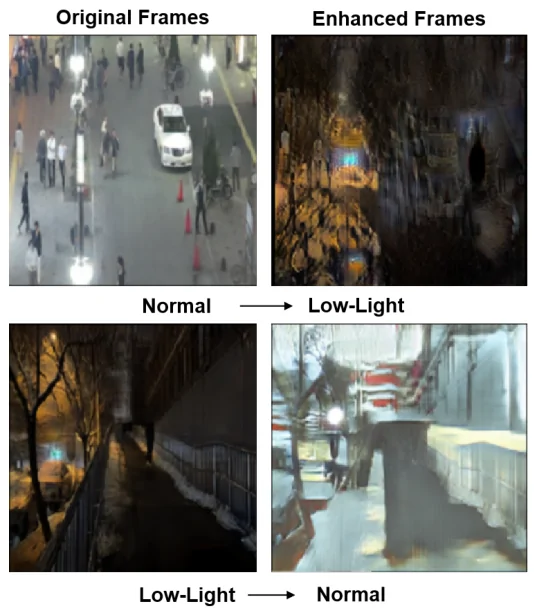
Low-light enhancement results obtained using CycleGAN. CycleGAN transforms normal-light images into low-light images through adversarial learning while preserving the underlying scene content.

CycleGAN introduces artifacts during nighttime image enhancement, particularly in video sequences with repetitive scenes, as shown in Figure 8. Its performance is highly sensitive to the number of training iterations. An insufficient number of iterations often results in inadequate detail learning and unrealistic scene appearance, whereas excessive iterations tend to amplify artifacts and introduce unnatural features. These issues degrade both the visual quality and the dataset’s utility for tasks like MOT. While CycleGAN shows potential for style transfer, its instability and artifact generation limit its effectiveness for high-quality nighttime enhancements in tracking datasets.

Unlike CycleGAN and Stable-Diffusion, the manually designed nighttime enhancement method effectively preserves ground truth (GT) annotations without introducing artifacts or inconsistencies, as illustrated in Figure 5. To further assess the statistical characteristics of the enhanced data, we compare the results of CycleGAN, Stable-Diffusion, and our method with two real nighttime datasets, the UniRTL nighttime RGBT dataset and the LLOT nighttime single-object tracking dataset. UniRTL exhibits relatively higher brightness and noise levels, whereas LLOT is substantially darker and has lower noise. These two datasets therefore provide complementary references for evaluating whether the enhanced data exhibit reasonable low-light characteristics.

As shown in Table 1, the data generated by all three enhancement methods fall within the overall statistical range defined by the two real nighttime datasets in terms of the BM, CM, and SNR, while their Noise_std values remain within or close to the range observed in the real nighttime data. This indicates that the three enhancement methods can produce images with broadly reasonable low-light statistical characteristics. However, statistical similarity alone does not guarantee visual quality or suitability for downstream tracking tasks. As discussed above, Stable-Diffusion may cause detail loss and lighting inconsistencies, whereas CycleGAN tends to introduce artifacts and unnatural structures. In comparison, LLMOT achieves a relatively low noise level while maintaining brightness and contrast characteristics within the range of real nighttime scenes, and more importantly, it preserves the original GT annotations without introducing obvious visual artifacts. Therefore, the manually designed enhancement method provides a more stable compromise between statistical characteristics, visual quality, and annotation consistency, making it more suitable for constructing a low-light MOT dataset.

In addition to image-level statistics, temporal consistency is an important property for video-based MOT datasets. Since LLMOT is generated from video sequences, we further evaluate whether the low-light transformation preserves inter-frame coherence. Following the evaluation protocol adopted in UniRTL, the warped SSIM and warped MAE are employed to measure temporal consistency after optical-flow compensation.

As shown in Table 2, compared with the original MOT17 videos, the generated low-light sequences exhibit a moderate decrease in the warped SSIM (0.9547 to 0.8027) and a slight increase in the warped MAE (0.0109 to 0.0142). This indicates that the low-light transformation introduces additional appearance variations while largely preserving temporal continuity. Although the temporal consistency is lower than that of naturally captured low-light videos in UniRTL, no severe temporal artifacts or frame-to-frame flickering are observed. Therefore, the generated low-light sequences maintain reasonable temporal coherence and remain suitable for low-light multi-object tracking research.

Table 3 compares LLMOT with representative MOT datasets in terms of task, modality, number of sequences, number of images/frames, annotation type, and lighting conditions. MOT17 and MOT20 are widely used RGB MOT benchmarks primarily collected under normal-light conditions, while UniRTL provides low-light MOT data with paired RGB and thermal modalities. In comparison, LLMOT is specifically constructed for RGB multi-object tracking in low-light environments and contains both labeled and unlabeled data. In terms of dataset scale, LLMOT is comparable to commonly used MOT benchmarks, with a size close to MOT17 and MOT20, although it is not the largest among existing datasets. This scale is reasonable for the target task because LLMOT is specifically designed to provide both labeled and unlabeled low-light RGB data, rather than to maximize the total number of frames. These characteristics distinguish LLMOT from existing MOT benchmarks and provide an appropriate data basis for semi-supervised low-light multi-object tracking.

**Figure 9:**
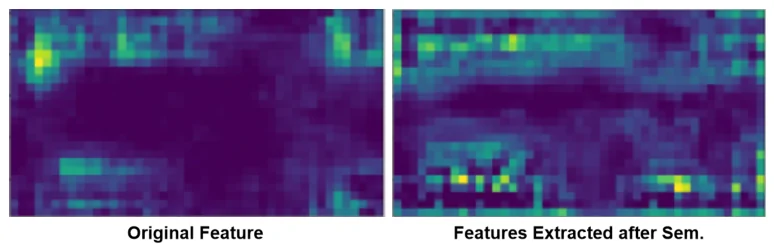
Visualization of appearance features extracted from the LLMOT dataset. Compared with models trained on normal-light data, the semi-supervised model (Sem.) extracts richer and more discriminative features from low-light images.

**Detection performance.** The performance of object detectors in low-light environments was evaluated under three distinct training settings. In the first setting, the detector was pretrained on normal lighting datasets and directly tested on low-light data without any additional training or fine-tuning (referred to as NL). In the second setting, the detector was initialized with the same pretrained model and fine-tuned on a low-light annotated dataset created by applying nighttime augmentation to the MOT17 dataset (referred to as LL). In the third setting, the detector was also initialized with the same pretrained model and fine-tuned on the entire LLMOT dataset using a semi-supervised algorithm, which incorporated both labeled and unlabeled low-light data (referred to as LLMOT). As illustrated in Figure 9, compared to the detector trained solely on normal lighting datasets (NL), the proposed approach demonstrates superior capability in extracting richer and more discriminative features from nighttime data, thereby significantly improving detection performance under low-light conditions.

The detection results are summarized in Table 4. From the table, it can be observed that fine-tuning the detector using the enhanced low-light annotated dataset (LL) significantly improves detection performance compared to directly using the normal lighting dataset (NL). This demonstrates the remarkable effectiveness of the enhanced low-light annotated dataset in improving object detection performance under low-light conditions. Furthermore, fine-tuning on the entire LLMOT dataset, which combines both labeled and unlabeled low-light data, leads to further performance improvements, achieving the best results. These findings validate the effectiveness of the proposed dataset in enhancing object detection performance in low-light environments and highlight the potential of semi-supervised learning methods in fully leveraging both labeled and unlabeled data.

**Tracking performance.** The tracking performance under low-light conditions is analyzed. Several representative tracking algorithms are compared, including motion-based association algorithms such as SORT [11], OC-SORT [16], and ByteTrack [15], as well as algorithms that combine motion and appearance, such as DeepSORT [12], MOTDT [61], Deep OC-SORT [17], GeneralTrack [40], UniSORT [42], and FeatureSORT [39].

The tracking results of each algorithm under low-light conditions are compared using detectors trained under three configurations: supervised training in normal lighting, supervised training in low-light, and semi-supervised training.

In detection-based tracking methods, choosing appearance-based or motion-based association methods significantly impacts detection effectiveness, which in turn influences subsequent association performance. As shown in Table 4 and Table 5, better detection performance leads to substantially improved tracking results. In low-light conditions, detection performance is weaker compared to normal lighting. Relying solely on motion cues for data association often leads to ID switches, especially in cases of pedestrian occlusions. Integrating motion and appearance cues helps mitigate this issue.

To further disentangle the contributions of detector training, semi-supervised learning, and association strategy, we analyze the results in Table 5 under controlled detector configurations. For the NL and LL settings, all compared trackers use the same YOLOX-x architecture and the same pretrained weights, with the LL detector further fine-tuned on the labeled low-light portion of LLMOT. Thus, under the same detector configuration, performance differences among different tracking methods mainly reflect the effects of their association strategies. The comparison between NL and LL shows that adapting the detector to low-light data substantially improves DetA and subsequently benefits the tracking performance of different association methods. Moreover, when the same association methods are equipped with the semi-supervised detector trained on LLMOT, further improvements are generally observed, particularly in DetA and MOTA, indicating the benefit of incorporating unlabeled low-light data to improve detection robustness. Importantly, all tracking methods in the semi-supervised setting use the same YOLOX-x detector trained with our semi-supervised strategy, providing a controlled comparison of their association performance. Under this setting, CRTrack achieves the highest HOTA, IDF1, and AssA among the compared methods, demonstrating that its additional performance gains are not solely attributable to the improved detector, but also result from the proposed association strategy. These results indicate that detector adaptation and semi-supervised learning primarily improve detection quality and robustness, while the proposed association strategy further enhances identity preservation and association accuracy.

**Figure 10:**
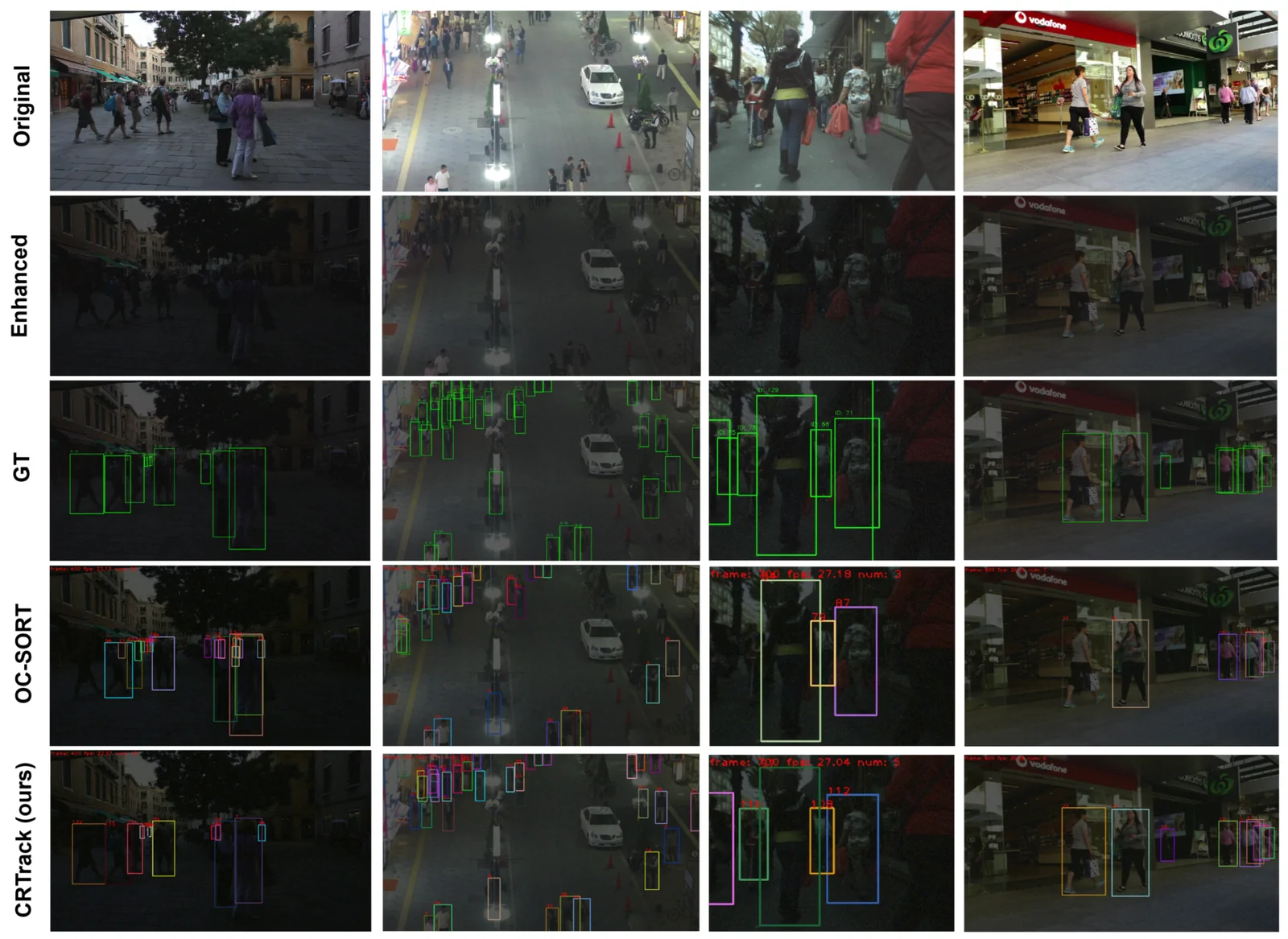
Example results on the LLMOT test set. Our CRTrack generates fewer tracking errors than OC-SORT [16].

Experimental results show that, compared to the baseline OC-SORT, the semi-supervised method CRTrack achieves significant performance gains on the DetA, MOTA, HOTA, IDF1 and AssA metrics, demonstrating the effectiveness of CRTrack. Figure 10 illustrates some example results on the LLMOT test set. The transformation from the original MOT17 data to the enhanced low-light data is shown in the first two rows. The ground truth (GT), OC-SORT, and CRTrack results are displayed in the following rows, enabling a direct comparison of tracking performance under low-light conditions. Furthermore, OC-SORT, which relies heavily on detection results to update tracks, shows limited tracking performance when detection quality is poor, particularly when transferring models trained in normal lighting to low-light environments. Semi-supervised training significantly mitigates this limitation by improving detection robustness. Although ByteTrack outperforms CRTrack on DetA and MOTA due to its staged matching strategy, which efficiently leverages low-confidence detection boxes to enhance recall, CRTrack demonstrates superior performance on HOTA and IDF1. By prioritizing track accuracy and robustness through semi-supervised learning, CRTrack adapts more effectively to low-light scenarios, generating higher-quality tracks with improved stability and association precision.

**Figure 11:**
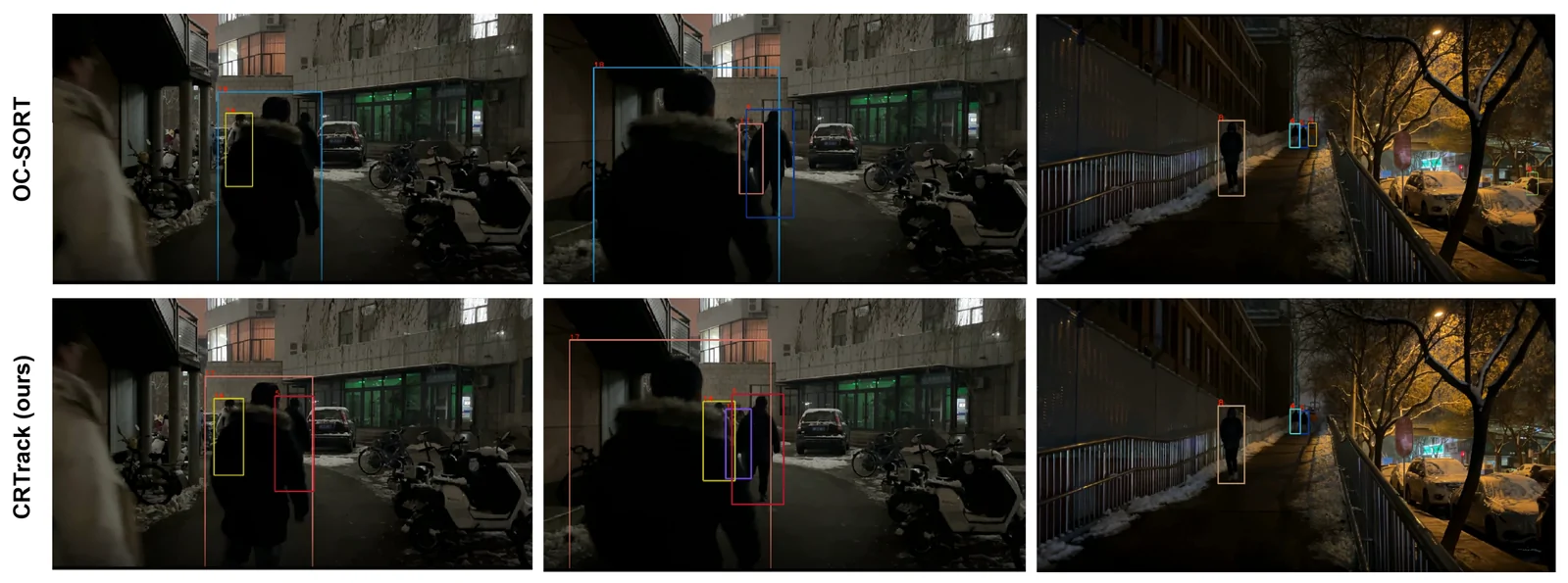
Tracking performance of OC-SORT and CRTrack in real low-light scenarios. Our CRTrack generates fewer tracking errors than OC-SORT.

In real low-light scenarios, we conducted a comparison test between OC-SORT and CRTrack. As shown in Figure 11, it illustrates the tracking performance of the two algorithms in complex nighttime environments. The first row shows the tracking results of OC-SORT, and the second row displays the results of CRTrack. OC-SORT exhibits issues such as object loss and less accurate tracking. In contrast, CRTrack demonstrates higher robustness and accuracy under the same conditions, maintaining stable tracking of objects and effectively identifying them even in complex backgrounds and low-light conditions.

**Evaluation on the real low-light UniRTL-MOT dataset.** To further evaluate the practical applicability and cross-dataset generalization of the proposed framework in real-world low-light environments, we conduct additional experiments on the UniRTL-MOT benchmark. To the best of our knowledge, UniRTL-MOT is the only publicly available pedestrian multi-object tracking dataset collected under real low-light conditions. Although it was originally designed for RGB-T tracking, the dataset also provides RGB video sequences, enabling the evaluation of RGB-based MOT methods in realistic low-light scenarios. Therefore, UniRTL-MOT provides an appropriate independent benchmark for assessing the generalization of models trained or fine-tuned using the proposed LLMOT dataset.

Considering the characteristics of the dataset, we evaluate all methods on the medium and high subsets. The low subset is excluded because most objects are nearly invisible in RGB images, making it more suitable for assessing the complementary benefits of thermal modalities rather than the capability of RGB-only tracking methods.

It should be noted that UniRTL-MOT is used solely for evaluation and is not involved in training or fine-tuning. For ByteTrack, OC-SORT, and FeatureSORT, we report results obtained using the original models (denoted as NL) as well as versions whose detectors are further fine-tuned on the proposed LLMOT dataset (denoted as LLMOT). Here, NL indicates that no LLMOT data are used during training, whereas LLMOT indicates detector fine-tuning using the proposed low-light dataset while keeping the original tracking framework unchanged. CRTrack adopts the complete semi-supervised framework proposed in this paper.

The results are presented in Table 6. Compared with the NL versions, the LLMOT-based variants consistently achieve better performance across different trackers, demonstrating that the low-light knowledge learned from LLMOT can be transferred to an independent real-world low-light dataset. Furthermore, CRTrack achieves the strongest overall association performance, indicating that the proposed framework effectively improves identity preservation and trajectory consistency in real-world low-light environments. These results further verify the effectiveness of the proposed approach and its ability to generalize across datasets and real-world low-light conditions.

### 5.3. Ablation Study

Ablation experiments are conducted to validate the effectiveness of our proposed improvements. The results are presented in Table 7. In the table, “Sem.” denotes the use of semi-supervised learning, “ASA” represents a consistent adaptive sampling assignment, and “ANU” indicates an adaptive network update. “App.” refers to the use of appearance features, while “SCD” represents the use of split cosine distance. All improvements are shown to contribute effectively to improved performance. As observed in Table 7, directly applying semi-supervised training yields second-best detection results. Introducing the ASA significantly improves the detection evaluation metric DetA. Incorporating the ANU further optimizes the model output, enhancing overall performance. Considering appearance features during association and employing the split cosine distance for calculation of similarity between tracking and observation features substantially improve IDF1, reduce ID switches, and enhance overall tracking performance.

Furthermore, experiments are conducted to analyze the weight factor $\lambda_{dis}$ for the ASA, as shown in Table 8. In this table, the values in the ASA column represent the specific weight values assigned to the ASA. Using OC-SORT as the association method, the best results are achieved when $\lambda_{dis}$ is set between 1 and 2. Due to the random data augmentation applied to the student network during semi-supervised learning, the training results exhibit some variability. Based on the analysis, $\lambda_{dis}=2$ is selected as the optimal value.

The effectiveness of the data augmentation methods was also evaluated, with the results presented in Table 9. Without any data augmentation, the model exhibited poor performance due to the lack of diversity in the training data, which limited its generalization ability. Gaussian augmentation improved performance slightly, indicating that random augmentations helped alleviate overfitting by introducing noise. In contrast, the proposed Night Aug (nighttime data augmentation), which was introduced in Section 3 and integrated into our semi-supervised network to enhance the student model, significantly boosted performance, with DetA reaching 60.532 and IDF1 increasing to 75.864. This improvement is due to Night Aug’s ability to simulate complex nighttime lighting conditions, which enhanced the robustness of the model in diverse scenarios.

In addition, the impact of the ratio between labeled and unlabeled data on model performance is analyzed, as shown in Table 10. When only labeled data is used (4:0 ratio), the model’s performance is suboptimal due to the limited labeled data, which restricts its generalization ability. As the proportion of unlabeled data increases, the model’s performance improves significantly, benefiting from the enhanced data diversity and robustness provided by the additional data. However, when the proportion of unlabeled data becomes too high (e.g., 1:3 ratio), performance slightly declines, likely due to noise in the unlabeled data reducing the quality of pseudo-labels and affecting the training process. Among all experiments, the 3:1 ratio of labeled to unlabeled data achieves the best balance, with DetA and IDF1 reaching 56.557 and 70.881, respectively. From a theoretical perspective, labeled data provides reliable supervision for generating high-quality pseudo-labels, while unlabeled data enhances data diversity through consistency regularization. The 3:1 ratio effectively combines these advantages, ensuring the quality of pseudo-labels while minimizing the negative impact of noise from the unlabeled data. This achieves an optimal balance between performance and robustness.

## 6. Conclusions

In this work, we propose the first pedestrian tracking dataset specifically designed for low-light environments. The dataset includes annotated data generated through enhancement techniques and unannotated low-light data collected from real-world scenes. This dataset serves as a critical resource for advancing research in pedestrian tracking in low-light conditions, enabling a more comprehensive evaluation and development of tracking algorithms in challenging environments.

We further investigate the task of MOT in dark scenes. To address the challenges of limited annotated data and the difficulties of manual annotation, we propose a semi-supervised MOT method tailored for low-light conditions. Based on the principle of consistency regularization, we design a “teacher–student” network framework that allows the model to learn robust features from both labeled and unlabeled data while reducing the impact of low-light-induced perturbations, thereby improving tracking performance in low-light conditions. Additionally, we introduce the ASA to suppress noisy or ambiguous samples under severe illumination degradation and enhance consistency between teacher and student networks. Furthermore, the ANU is developed to update the teacher network more flexibly.

Our work addresses the challenges posed by limited annotated data, while enhancing tracking capabilities in low-light environments. By leveraging semi-supervised learning, the proposed method improves feature robustness and tracking accuracy, providing a valuable framework for reliable MOT in challenging low-light conditions.

Although the proposed framework achieves promising results, the reliability of pseudo-label generation may still be affected by severe low-light degradation, leaving room for further improvement in extremely challenging scenes. In addition, the current LLMOT data are partitioned at the frame level within each video sequence, which may introduce potential temporal and scene leakage between the training, validation, and test sets, making the corresponding evaluation potentially optimistic compared with a strict video-level partitioning protocol. Although the effectiveness of the proposed method has also been demonstrated on the independent real-world low-light multi-object tracking dataset UniRTL-MOT, stricter video-level partitioning and scene-independent evaluation remain necessary. In future work, we plan to explore more robust pseudo-label generation strategies, adopt strict video-level data partitioning protocols, and construct and annotate more real-world low-light MOT test data to further evaluate the practical robustness and generalization capability of the proposed method and expand the LLMOT dataset.

## Figures and Tables

**Table 1:** Quantitative comparison of the brightness mean (BM), noise standard deviation (Noise_std), contrast measure (CM), and signal-to-noise ratio (SNR) among data generated by three low-light enhancement methods and two real nighttime datasets. The BM, Noise_std, and CM are computed on 8-bit images (0–255), and the SNR is reported in dB.

Dataset	BM	Noise_Std	CM	SNR
UniRTL	78.9089	30.8451	[69.14, 69.88, 70.75]	−1.59
LLOT	16.0229	18.1044	[15.62, 15.81, 16.51]	2.82
MOT17 (CycleGAN)	40.2	26.4	[38.7, 40.1, 41.0]	1.35
MOT17 (Stable-Diffusion)	39.8	22.6	[38.1, 39.6, 40.5]	1.82
LLMOT (Ours)	41.7075	19.7284	[40.12, 41.79, 42.15]	2.16

**Table 2:** Temporal consistency comparison using optical-flow-compensated metrics. An upward arrow (↑) indicates that higher values are better, whereas a downward arrow (↓) indicates that lower values are better.

Dataset	Warped SSIM ↑	Warped MAE ↓
UniRTL	0.9850	0.0037
Original MOT17	0.9547	0.0109
LLMOT (Ours)	0.8027	0.0142

**Table 3:** Statistics and comparison of representative MOT datasets under different lighting conditions. L and U denote labeled and unlabeled data, respectively.

Dataset	Task	Modality	Sequences	Images/Frames	Annotation	Lighting
MOT17	MOT	RGB	21	11,235	Labeled	Normal
MOT20	MOT	RGB	8	13,410	Labeled	Normal
UniRTL	MOT	RGB + Thermal	50	25,077	Labeled	Low-light
**LLMOT (Ours)**	MOT	RGB	7 + 10	11,580	5316 L + 6264 U	Low-light

**Table 4:** Detection performance under different training data settings. NL denotes training on the normal-light set, LL denotes fine-tuning on the enhanced low-light annotated set, and LLMOT denotes fine-tuning on the full mixed low-light set containing both labeled and unlabeled data. Best results are shown in bold.

Training Data	mAP	AP50	AP75	${\mathrm{AP}}_{S}$	${\mathrm{AP}}_{M}$	${\mathrm{AP}}_{L}$	mAR	${\mathrm{AR}}_{S}$	${\mathrm{AR}}_{M}$	${\mathrm{AR}}_{L}$
NL	46.5	73.6	52.3	7.1	36.5	60.3	50.7	8.4	40.9	65.0
LL	52.8	79.1	59.8	12.7	41.9	66.8	57.9	18.3	48.8	70.7
LLMOT	**54.6**	**82.0**	**61.5**	**14.6**	**44.9**	**66.9**	**59.4**	**25.2**	**51.4**	**70.9**

**Table 5:** Tracking performance of different methods in low-light environments. The upper and lower sections report supervised and semi-supervised results, respectively. NL denotes training on the normal-lighting set, LL denotes fine-tuning on the enhanced low-light annotated set, and LLMOT denotes fine-tuning on the mixed low-light set containing labeled and unlabeled data. Sem. (ours) denotes our semi-supervised detector trained on LLMOT. Bold and underlined values indicate the best and second-best results, respectively.

Method	Association	Detection	Training Data	DetA	MOTA	HOTA	IDF1	AssA
SORT [11]	Motion	YOLOX	NL	44.534	52.594	51.233	63.293	59.208
	Motion	YOLOX	LL	55.798	64.550	58.059	69.771	60.908
OC-SORT [16]	Motion	YOLOX	NL	44.470	52.730	50.527	61.459	57.660
	Motion	YOLOX	LL	55.804	65.144	57.754	68.873	60.233
ByteTrack [15]	Motion	YOLOX	NL	50.742	61.215	55.638	68.493	61.317
	Motion	YOLOX	LL	59.561	70.52	60.381	72.847	61.616
DeepSORT [12]	Motion + Appearance	YOLOX	NL	48.694	58.547	53.677	66.257	59.460
	Motion + Appearance	YOLOX	LL	58.263	68.124	58.85	70.031	60.059
MOTDT [61]	Motion + Appearance	YOLOX	NL	48.861	58.514	53.777	65.892	59.493
	Motion + Appearance	YOLOX	LL	58.633	67.588	58.924	69.706	59.779
Deep OC-SORT [17]	Motion + Appearance	YOLOX	NL	47.886	57.152	53.581	65.694	60.224
	Motion + Appearance	YOLOX	LL	58.272	69.282	60.608	74.265	63.335
GeneralTrack [40]	Motion + Appearance	YOLOX	NL	50.838	61.313	55.635	68.452	61.671
	Motion + Appearance	YOLOX	LL	59.729	70.196	60.381	72.959	61.876
UniSORT [42]	Motion + Appearance	YOLOX	NL	45.860	54.880	52.150	63.980	59.050
	Motion + Appearance	YOLOX	LL	56.880	66.320	58.620	70.120	60.980
FeatureSORT [39]	Motion + Appearance	YOLOX	NL	49.786	58.944	54.381	66.297	60.132
	Motion + Appearance	YOLOX	LL	58.897	68.694	59.357	70.663	60.584
SORT [11]	Motion	Sem. (ours)	LLMOT	57.285	66.968	59.908	71.655	63.068
OC-SORT [16]	Motion	Sem. (ours)	LLMOT	57.190	66.883	59.978	72.171	63.256
ByteTrack [15]	Motion	Sem. (ours)	LLMOT	**60.896**	**71.624**	61.816	73.614	63.506
DeepSORT [12]	Motion + Appearance	Sem. (ours)	LLMOT	59.715	70.360	62.041	74.674	64.931
MOTDT [61]	Motion + Appearance	Sem. (ours)	LLMOT	60.016	70.583	61.346	74.039	63.183
Deep OC-SORT [17]	Motion + Appearance	Sem. (ours)	LLMOT	60.257	71.052	61.832	74.799	63.862
GeneralTrack [40]	Motion + Appearance	Sem. (ours)	LLMOT	60.461	71.371	62.249	74.982	64.943
UniSORT [42]	Motion + Appearance	Sem. (ours)	LLMOT	58.320	68.020	60.830	73.620	64.060
FeatureSORT [39]	Motion + Appearance	Sem. (ours)	LLMOT	60.269	71.131	62.165	75.358	64.932
CRTrack (ours)	Motion + Appearance	Sem. (ours)	LLMOT	60.532	71.544	**62.472**	**75.864**	**64.976**

**Table 6:** Performance comparison on the RGB subset of the UniRTL-MOT dataset. NL denotes detectors trained on normal-light data, while LLMOT denotes detectors fine-tuned using the proposed low-light dataset. Best results are shown in bold.

Method	Training Data	DetA	MOTA	HOTA	IDF1	AssA
ByteTrack	NL	47.446	59.375	50.887	68.089	55.274
	LLMOT	**48.102**	**60.600**	51.344	68.302	55.528
OC-SORT	NL	43.277	54.060	47.425	64.917	52.426
	LLMOT	44.194	55.506	48.625	66.000	53.944
FeatureSORT	NL	45.729	56.937	49.845	67.587	55.497
	LLMOT	46.332	57.431	50.154	68.097	56.364
CRTrack (Ours)	LLMOT	47.034	58.621	**52.181**	**70.284**	**58.352**

**Table 7:** Ablation study on semi-supervised learning (Sem.), consistent adaptive sampling assignment (ASA), adaptive network update (ANU), appearance feature (App.), and split cosine distance (SCD). A checkmark indicates that the corresponding component is enabled. Best results are shown in bold.

OC-SORT	Sem.	ASA	ANU	App.	SCD	DetA	MOTA	HOTA	IDF1	AssA
✓						55.804	65.144	57.754	68.873	60.233
✓	✓					55.729	65.923	58.428	70.290	61.585
✓	✓	✓				56.557	66.081	59.011	70.881	61.993
✓	✓	✓	✓			57.190	66.883	59.978	72.171	63.256
✓	✓	✓	✓	✓		60.257	71.052	61.832	74.799	63.862
✓	✓	✓	✓	✓	✓	**60.532**	**71.544**	**62.472**	**75.864**	**64.976**

**Table 8:** Ablation study on ASA weights. Best results are shown in bold.

ASA	DetA	MOTA	HOTA	IDF1	AssA
0	55.729	65.923	58.428	70.290	61.585
1	55.747	65.942	58.807	**71.287**	**62.348**
2	**56.557**	**66.081**	**59.011**	70.881	61.993
3	55.157	64.622	57.613	68.763	60.536

**Table 9:** Ablation study of augmentation methods. Best results are shown in bold.

Aug	DetA	MOTA	HOTA	IDF1	AssA
No	59.647	71.328	61.843	75.273	64.459
Gaussian	59.961	71.391	62.178	75.439	64.686
Night	**60.532**	**71.544**	**62.472**	**75.864**	**64.976**

**Table 10:** Ablation study on the ratio of labeled and unlabeled images in each batch (labeled:unlabeled). Best results are shown in bold.

Ratio	DetA	MOTA	HOTA	IDF1	AssA
4:0	55.804	65.144	57.754	68.873	60.233
1:3	53.947	64.091	56.361	67.662	59.427
2:2	56.213	65.682	58.504	69.653	60.869
3:1	**56.557**	**66.081**	**59.011**	**70.881**	**61.993**

## Data Availability

The raw data supporting the conclusions of this article will be made available by the authors on request.
